# A Multi-Site, International Audit of Malnutrition Risk and Energy and Protein Intakes in Patients Undergoing Treatment for Head Neck and Esophageal Cancer: Results from INFORM

**DOI:** 10.3390/nu14245272

**Published:** 2022-12-10

**Authors:** Lisa Martin, Merran Findlay, Judith D. Bauer, Rupinder Dhaliwal, Marian de van der Schueren, Alessandro Laviano, Adrianne Widaman, Vickie E. Baracos, Andrew G. Day, Leah M. Gramlich

**Affiliations:** 1 Department of Medicine, University of Alberta, Edmonton, AB T6G 2G3, Canada; 2 Cancer Services, Royal Prince Alfred Hospital, Camperdown, NSW 2050, Australia; 3 Chris O’Brien Lifehouse, Camperdown, NSW 2050, Australia; 4 Department of Nutrition, Dietetics and Food, Monash University, Clayton, VIC 3800, Australia; 5 Canadian Nutrition Society, Ottawa, ON K0G 1J1, Canada; 6 Human Nutrition and Health, Wageningen University and Research, 6700 AA Wageningen, The Netherlands; 7 Department of Translational and Precision Medicine, Sapienza University of Rome, I-00185 Rome, Italy; 8 Department of Nutrition, Food Science and Packaging, San Jose State University, San Jose, CA 95192, USA; 9 University of California Davis Medical Center, Sacramento, CA 94558-5004, USA; 10 Cross Cancer Institute, Department of Oncology, University of Alberta, 11560 University Avenue, Edmonton, AB T6G 1Z2, Canada; 11 Department of Public Health Sciences, Queen’s University, Kingston, ON K7L 2V7, Canada; 12 Royal Alexandra Hospital, Division of Gastroenterology, Department of Medicine, University of Alberta, Edmonton, AB T5H 3V9, Canada

**Keywords:** malnutrition, nutrition care, head and neck cancer, esophageal cancer, foregut tumors, nutrition risk, patient-generated subjective global assessment, nutrition support, enteral nutrition, energy intake

## Abstract

Patients with foregut tumors are at high risk of malnutrition. Nutrition care focuses on identifying individuals at risk of malnutrition and optimizing nutrient intake to promote the maintenance of body weight and lean body mass. This multi-center prospective, longitudinal study audited nutrition care practices related to screening for risk of malnutrition (Patient-Generated Subjective Global Assessment Short Form; PG-SGA SF), and nutrition interventions prescribed (route; adequacy of energy and protein intakes). Audits occurred at four time periods: baseline (before treatment) and at 2, 4, and 6 months after starting cancer treatment; 170 patients (esophageal (ESO; *n* = 51); head and neck (HN; *n* = 119)) were enrolled. Nutrition risk (PG-SGA SF score ≥ 4) was prevalent at every time period: HN (baseline: 60%; 6 months 66%) and ESO (77%; 72%). Both groups had significant (*p* < 0.001) weight losses over the 6 month audit period (HN = 13.2% ESO = 11.4%). Enteral nutrition (EN) was most likely to be prescribed at 2 months for HN and at 4 and 6 months for ESO. Target prescribed energy and protein intakes were not met with any nutrition intervention; although adequacy was highest for those receiving EN. Nutrition care practices differed for HN and ESO cancers and there may be time points when additional nutrition support is needed.

## 1. Introduction

Patients with foregut tumors, located in the head, neck and esophagus, are at high risk of malnutrition due to gastrointestinal symptoms and side effects incurred from lengthy, multi-modal cancer treatments [1,2,3]. Patients often experience weight loss and problems eating before they start treatment, which are further exacerbated during treatment, and are associated with poor clinical outcomes [4,5,6,7,8,9,10,11,12,13,14,15]. The hallmark of cancer-associated malnutrition is involuntary weight loss, which is characterized by the loss of skeletal muscle (with or without fat loss) as a result of negative energy and protein balance driven by a variable combination of reduced food intake and metabolic alterations. [16] In patients with foregut tumors, reductions in food intake are multifactorial and likely the predominant driver of weight loss [5,17]. Symptoms such as loss of appetite, physical obstruction (dysphagia, pain), and treatment related side effects (nausea, mucositis, dysgeusia, xerostomia) conspire to severely impair food intake [5,6,7,8,11,18,19]. Metabolic alterations on the other hand, are suggested to include inflammation, increased energy expenditure, and excess catabolism in response to the tumor and cancer treatments [3,16,20,21]. Given the multifactorial nature of cancer-associated malnutrition, multimodal treatment approaches are recommended, which include optimal nutrition care, pain and symptom management, exercise, and modulation of inflammation [3,20,22,23,24,25]. From a nutrition care perspective a main goal for preventing and managing cancer-associated malnutrition is to optimize nutrient intake to promote the maintenance of body weight and lean body mass [3,20,22,23].

The best-available evidence to guide nutrition care practices for cancer has been synthesized in evidence-based guidelines (EBGs) from Australia, Europe, North America and the United Kingdom [22,23,24,26,27,28,29,30,31,32,33,34]. Optimizing nutrition care delivery has been demonstrated to have a positive impact on clinical, cost, and patient-centered outcomes. EBGs address different aspects of the nutrition care process which comprises three domains: (1) Appropriate Access to Care (Nutrition Screening and Assessment); (2) Quality Nutrition Care (Goals, Prescription, Implementation); and (3) Nutrition Evaluation and Monitoring (Measure and Evaluate Outcomes) [35,36]. In the first report from the International Nutrition Audit in FORegut TuMours (INFORM) Study, Findlay et al. [37]. described adherence of current real-world nutrition care practices to EBGs. Overall, participating sites had good adherence (>80%) to guidelines pertaining to routine malnutrition screening, nutrition assessment, and prescriptions for energy and protein intakes. However, guidelines pertaining to use of validated screening and assessment tools, regular dietetic consultation, and initiation of nutrition support had moderate (60 to 80%) to poor adherence (<60%) levels. This prospective, longitudinal study aims to determine the translation of EBGs into nutrition care practices related to screening for risk of malnutrition, the route of nutrition support used, and adequacy of energy and protein intakes over time in patients with esophageal and head and neck cancers.

## 2. Materials and Methods

### 2.1. Study Design and Patients

This was a multicenter, prospective, registry design that included a baseline audit of nutrition care practices, followed by repeated audit cycles from up to 6 months from the first time a patient was introduced to the cancer care system. Audits of nutrition care practices occurred across four time periods. The baseline period commenced with patients’ first date of contact with their respective cancer care system, and included nutrition practices captured ±30 days from this date; the other audit periods were defined using the baseline period as the reference: 2 months (31–90 days after baseline), 4 months (91–150 days) and 6 months (151–210 days).

Adult patients (≥18 years of age) newly diagnosed with head and neck (HN) or esophageal (ESO) cancers scheduled to receive cancer treatment (any modality) were enrolled. Patients were excluded (i.e., not enrolled) if there was no treatment plan due to patient’s imminent death, or if their Eastern Cooperative Oncology Group (ECOG) Performance Status Score was ≥4. Participating sites obtained ethics approval from their local research ethics boards, informed consent was obtained from all subjects involved in the study.

### 2.2. Data Collection

Data were collected from patients at 11 cancer care centers in Canada (*n* = 6), Australia (*n* = 2), Italy (*n* = 1), The Netherlands (*n* = 1), and the United States (US) (*n* = 1). Sites were academic hospitals that included a patient care dietitian or a nutrition-focused health care provider (e.g., Clinical Nutritionist, or Nutrition Delegate) who collected the data; these individuals are referred to as a dietitian throughout.

Data were collected longitudinally from patients using quota sampling (from 2016–2018) and entered into online case report forms using Research Electronic Data Capture (REDCap) hosted by the University of Alberta, Canada. Each site aimed to collect data on up to 20 patients. Case report forms were completed by a dietitian. Patient demographics, cancer diagnosis and stage (AJCC staging v.8), and treatment modality (surgery, chemotherapy, radiation therapy) were collected. Patient heights and weights were recorded and BMI [body weight (kg)/height (m^2^)] and % weight change before treatment [((current weight (kg) − weight 6 months ago (kg))/weight 6 months ago (kg)) × 100] were calculated. Data related to nutrition care practices were collected including screening for risk of malnutrition, nutrition prescriptions and interventions used.

### 2.3. Screening for Risk of Malnutrition

Evidence-based guidelines recommend screening for risk of malnutrition using a validated tool at baseline, which is then repeated at regular intervals throughout treatment [22,24,28,30,33]. The PG-SGA SF is a practicable malnutrition risk screening tool validated for patient report in oncology settings [38,39,40]. The PG-SGA SF was initiated as part of the study protocol to create consistency between sites and to facilitate longitudinal comparisons of malnutrition risk across sites. Dietitians administered a paper version of the PG-SGA SF and responses were self-reported by the patient. The PG-SGA SF is a single-page questionnaire comprising questions about changes in body weight (Box 1), food intake (Box 2), nutrition impact symptoms (Box 3), and activity and function level (Box 4). Points from the 4 boxes are summed to yield a total score (range 0 to 37; higher scores indicate higher nutrition risk), which are categorized according to pre-specified nutrition triage recommendations: scores 0–1 indicate no intervention is required at this time but re-assessment on routine and regular basis is needed during treatment; scores 2–3 recommend patient & family education by dietitian, nurse, or other clinician with pharmacologic intervention as indicated by symptom survey (Box 3) and lab values as appropriate; scores 4–8 the individual requires intervention by dietitian, in conjunction with nurse or physician as indicated by symptoms (Box 3); scores ≥ 9 indicate a critical need for improved symptom management and/or nutrient intervention options. In this study, a score ≥ 4 identified individuals at risk of malnutrition [38]. When more than one PG-SGA SF was completed within the same time period for a given patient, which would occur if a patient had multiple follow-ups with a dietitian in a given time period, the form closest to the center of the time period was used (i.e., day 0, 60, 120 or 180) for analysis.

### 2.4. Nutrition Prescription and Intervention

For each patient, the dietitian provided nutrition prescriptions, which included estimated energy (kcal/kg body weight/day) and protein (g pro/kg body weight/day) requirements based on the individual dietitians’ assessment of the patient, and a patient-centered nutrition intervention was documented. Nutrition prescriptions, route of nutrition, and timing of initiation according to local practices were collected. Route of nutrition refers to the manner in which nutrition was delivered to an individual, which was categorized as oral intake only (oral only), oral intake with use of oral nutritional supplements (oral with ONS), or use of enteral nutrition with or without oral intake (EN; included nasogastric, gastrostomy, jejunostomy feeding tubes). Patients were categorized according to a route of nutrition if they were ever prescribed that route within the 6 month study period. There were only 4 patients prescribed parenteral nutrition (PN), and in each case they were also receiving EN and were therefore categorized as using EN. Adequacy of energy and protein intakes were calculated as a percent of the prescription received relative to what was prescribed by the dietitian [(estimated energy or protein intake/amount energy or protein prescribed) × 100].

### 2.5. Statistics

Analyses were performed separately by cancer type: HN and ESO groups. Results are presented descriptively by time period using medians and quartiles [Q1, Q3], counts and percentages, or means (±standard deviation) where appropriate. All model-based estimates are reported as estimated means and standard errors (SE).

Our study design was prospective longitudinal; PG-SGA SF data, amount of energy and protein prescribed, estimated energy and protein intakes, and route of nutrition were documented repeatedly over the course of the study. As is typical with longitudinal data, we had unbalanced data due to missing data points (i.e., missing at random) given that this study was developed based on clinical practice where data is collected at irregular, subject-specific intervals.

To assess statistical significance of outcomes over time, regression models recommended for longitudinal data were used. For continuous outcomes (PG-SGA SF scores, % weight change, adequacy of energy and protein intakes), the linear mixed effects model estimated by restricted maximum likelihood as implemented in the MIXED procedure of SAS was used. The mixed model estimates the mean given all people remaining in the dataset. When comparing values over time, we included patient as a random effect (i.e., missing at random) and time period as a categorical independent variable, except for weight loss where we used the random coefficients model with month treated as a random linear slope which could vary along with the intercept between individuals. When comparing PG-SGA SF scores, adequacy of intake, or weight change by route of nutrition, we pooled across time periods including time period and route of nutrition as categorical independent variables. To compare route of nutrition over time we used ordinal regression to model intensity of nutrition support (EN > oral with ONS > oral intake only) as the dependent variable accounting for in patient dependence by using generalized estimating equations clustered by patient as implemented in the GENMOD procedure of SAS.

SAS Version 9.4 (SAS Institute Inc., Cary, NC, USA) was used for all analysis. All *p*-values were two-sided without adjustment for multiplicity of tests. A ***p*-value < 0.05** was indicative of statistical significance.

## 3. Results

A total of 170 patients (*n* = 119 HN; *n* = 51 ESO) were enrolled (Table 1), although slightly fewer patients were used for particular parts of the analysis due to missing data (Table S1).

### 3.1. What Did the Audit Tell Us about Risk of Malnutrition?

#### 3.1.1. HN Cancers

At baseline, 5% of patients were underweight (BMI < 18.5), and 25% were obese (BMI ≥ 30.0). In the 6 months prior to enrolling in the study, patients had lost a mean (±SD) of 3.8 ± 7.5% of their body weight. According to the PG-SGA SF, the percentage of patients at risk of malnutrition (score ≥ 4) was high across all study periods (60% at baseline, 86% at 2 months, 75% at 4 months, and 66% at 6 months; Table 2).

**Figure 1:**
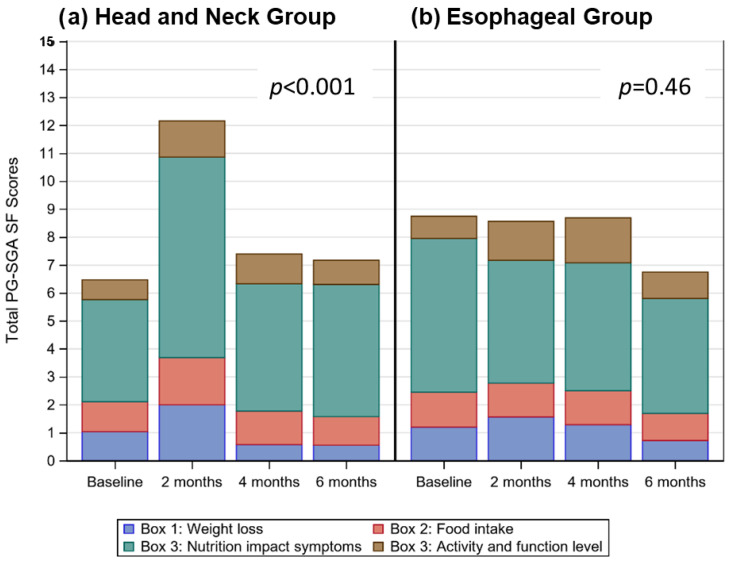
Stacked bar charts for (**a**) and (**b**) display the distribution of mean total PG-SGA SF scores over time, highlighting the contribution of weight loss (Box 1—blue), food intake (Box 2—red), nutrition impact symptoms (Box 3—teal), and activity and function level scores (Box 4—brown) to the total score at each time period.

PG-SGA SF scores were higher (*p* < 0.001) from baseline at 2 months, due to increased nutrition impact symptoms (Figure 1a). Patients lost weight (*p* < 0.001) from baseline to 6 months; based on linear mixed modelling patients lost an estimated average of 2.2% (0.2) per month, equating to an estimated weight loss of 13.2% over the 6 month audit period.

#### 3.1.2. ESO Cancers

At baseline 2% of patients were underweight (BMI < 18.5), and 31% were obese (BMI ≥ 30.0). In the 6 months prior to enrolling in the study, patients had lost a mean (±SD) of 5.1 ± 7.2% of their body weight. According to the PG-SGA SF, the percentage of patients at risk of malnutrition was high, and remained unchanged (*p* = 0.46; Figure 1b) across all study periods (77% at baseline, 71% at 2 months, 85% at 4 months, 72% at 6 months; Table 2). Patients lost weight (*p* < 0.001) from baseline to 6 months; based on linear mixed modelling patients lost an estimated average of 1.9% (0.2) per month, equating to an estimated weight loss of 11.4% over the 6 month audit period.

### 3.2. What Did the Audit Tell Us about the Timing and Type of Nutrition Route Used?

**Figure 2:**
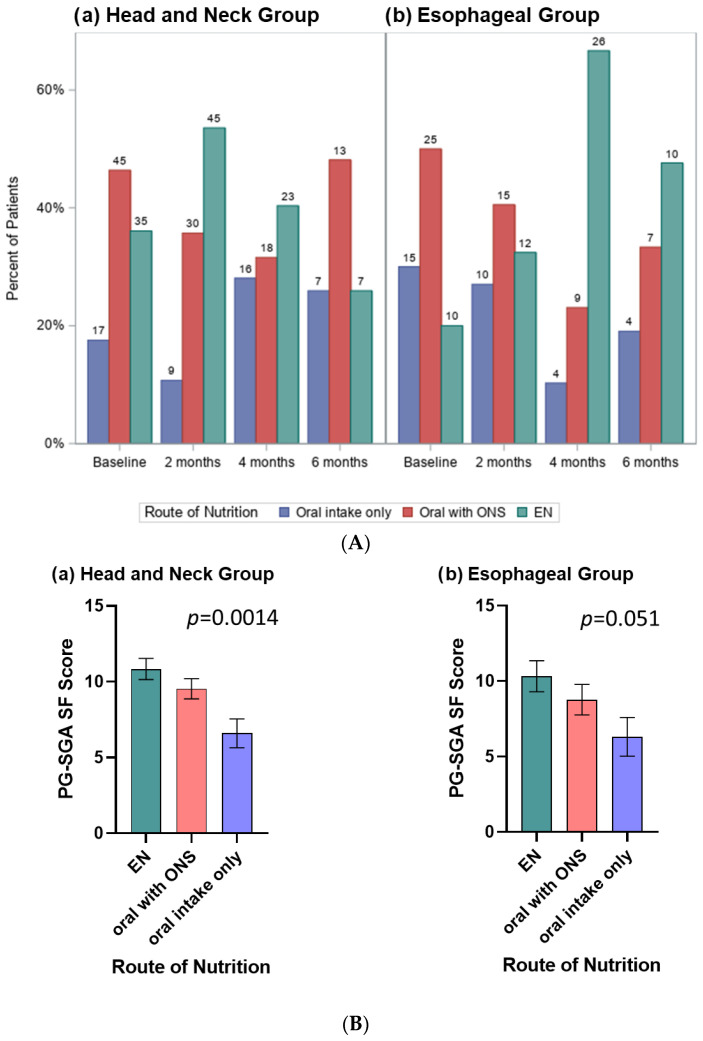
(**A**) Clustered bar charts display the frequency of route of nutrition used by time period for the(**a**) Head and Neck Group and (**b**) Esophageal Group; numbers above each bar indicate the number of patients. (**B**) Bar charts display the linear mixed model estimated mean (SE) PG-SGA SF scores for patients with (**a**) Head and Neck Group and (**b**) Esophageal Group according to route of nutrition, controlled for time period. The trend in both groups was for patients receiving EN to have the highest PG-SGA SF scores, followed by oral with ONS, and then the oral only route with the lowest scores.

The relative frequency of nutrition routes used varied over time for HN (*p* = 0.0038) and ESO (*p* = 0.0008) cancers (Figure 2A). The ESO group were over twice as likely to use EN at 4 months (67%) and 6 months (48%) compared to baseline (20%) and 2 months (32%). The trend, although weaker, was opposite in the HN group who were more likely to use EN at 2 months (54%) compared to baseline (36%), 4 months (40%) and 6 months (26%).

When evaluated using linear mixed models, after controlling for time period, PG-SGA SF scores varied across route of nutrition for HN (*p* = 0.0014) and trended to significance for ESO (*p* = 0.051) cancers (Figure 2B). The trend in both groups was for patients receiving EN to have the highest PG-SGA SF scores, followed by oral with ONS, and oral only route with the lowest scores.

### 3.3. What Did the Audit Tell Us about the Adequacy of Energy and Protein Intakes According to Nutrition Route?

**Figure 3:**
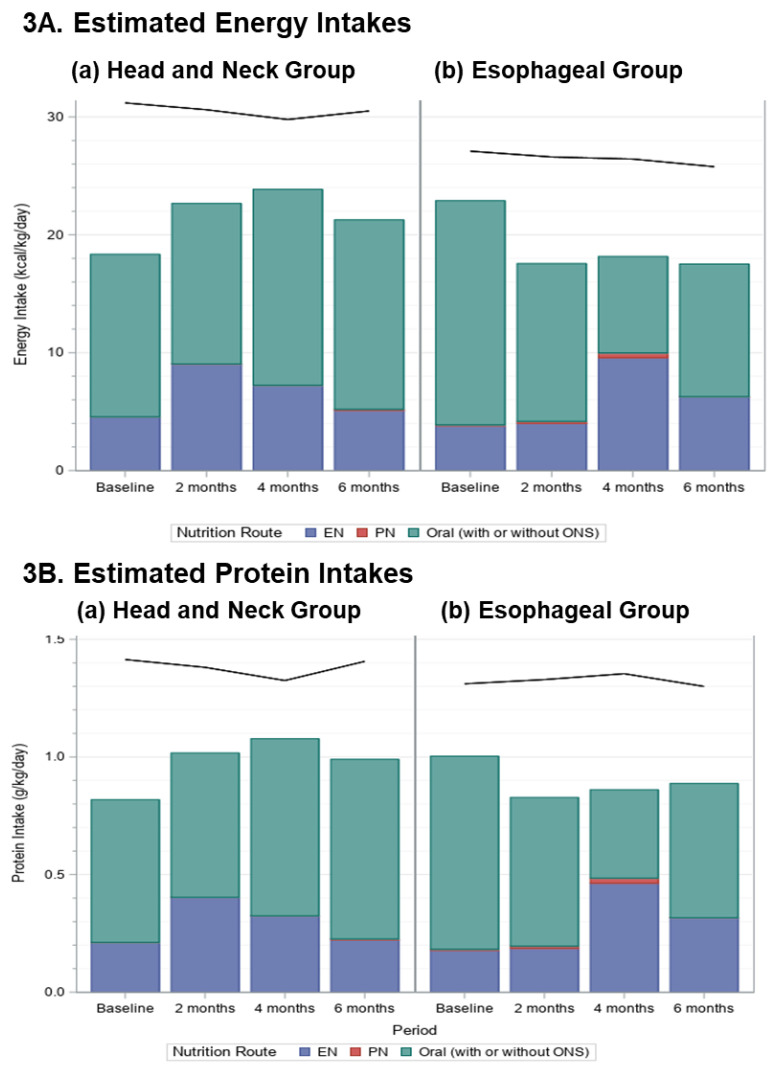
Stacked bar charts display the mean (**A**) energy (kcal/kg/d) and (**B**) protein (g/kg/d) intakes by route of nutrition used (Enteral Nutrition (EN)—blue; Parenteral Nutrition (PN)—red; Oral intake (with or without ONS)—teal); for patients with HN and ESO cancers for each time period; the black lines indicate the average nutrient prescription.

The mean (±SD) prescriptions, pooled across time periods, for energy was 30.4 ± 4.9 kcal/kg/day (min 20.8–max 41.7) and for protein was 1.4 ± 0.3 (0.7–2.1) g/kg/day for HN group (*n* = 88). The mean prescriptions, pooled across time periods, for energy was 26.6 ± 4.2 kcal/kg/day (19.0–35.7) and for protein was 1.3 ± 0.2 (0.8–2.0) g/kg/day for ESO group (*n* = 50), and did not change over time (black lines in Figure 3).

For the HN group, adequacy of energy (*p* = 0.14) and protein (*p* = 0.064) intakes did not change over time (Figure 3). For the ESO group, adequacy of energy intake declined (*p* = 0.0059) due to a reduction after baseline, but protein intake although decreased, was not affected (*p* = 0.082). It is notable that energy and protein intakes were below the prescribed amounts in all periods, and that, except for at 4 months in the ESO group, most of the nutrition was received orally (with or without ONS; Figure 3).

**Figure 4:**
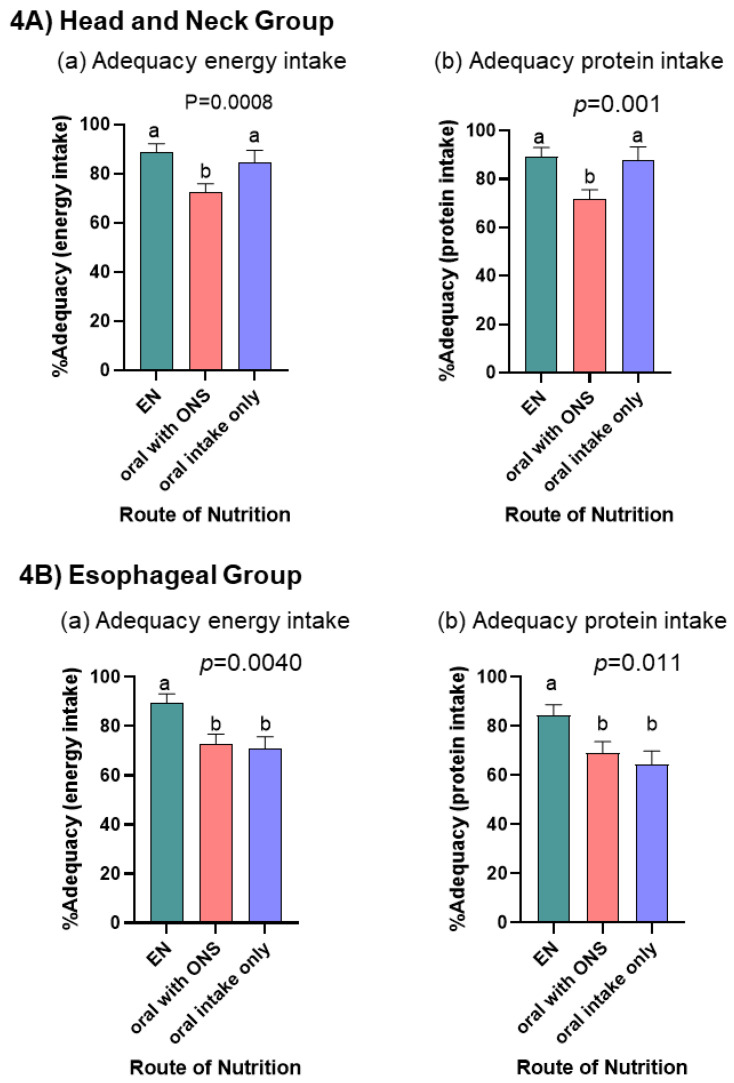
Bar charts display the linear mixed model estimated mean (SE) % adequacy [(estimated intake/amount prescribed) × 100%] for energy and protein intake for the (**A**) Head and Neck Group (**a**,**b**) and (**B**) Esophageal Group (**a**,**b**) cancers. Each bar represents the within person estimated mean (SE) averaged across the different nutrition routes over time for an individual with an average PG-SGS SF score. *p*-values represent significant differences in % adequacy by route of nutrition adjusted for time period and PG-SGA SF score. ^a,b^ indicate differences (*p* < 0.05) between nutrition routes used.

When linear mixed models were adjusted for PG-SGA SF score and time period, there was a difference in adequacy of energy and protein intakes by route of nutrition for both the HN (*p* = 0.0008; *p* = 0.001) and ESO (*p* = 0.004; *p* = 0.011) groups (Figure 4). HN cancers had lowest adequacy of energy and protein intake with the nutrition route oral with ONS when compared to EN and oral only routes. ESO cancers had highest adequacy of energy and protein intake with the nutrition route EN when compared to oral with ONS or oral only routes. Although energy and protein targets were not met in any time period, linear mixed models show for HN patients, as energy and protein adequacy increased, patients experienced less weight loss (*p* < 0.001), when controlling for nutrition route. For ESO patients, neither the amount of energy nor protein received (*p* = 0.97; *p* = 0.84), nor route of nutrition (*p* > 0.1) were associated with weight loss. 

## 4. Discussion

In this study, we prospectively evaluated malnutrition risk, nutrition prescriptions and intervention data from foregut tumor patients undergoing cancer treatment at multiple international cancer centers over a period of 6 months. Data from the PG-SGA SF revealed that patients were at risk of malnutrition throughout the study period, and highlighted points in time along the treatment trajectory where EN and ONS were more likely to be prescribed. For patients with HN cancers this occurred at 2 months, and for ESO patients it was at 4 and 6 months. Although patients receiving EN had the highest energy and protein intakes, target intakes were not met with any nutrition route, in any time period, and weight loss was not attenuated. Over the 6 month audit period, HN and ESO patients experienced significant weight losses of 13.2% and 11.4%, respectively. PN was not considered in this study as it was only prescribed concurrently with EN for 4 patients (2.5%), likely because patients were treated in an outpatient setting, and in many centers PN is only prescribed in inpatient settings. In this study, energy and protein intakes were suboptimal even when patients received ONS or EN. This study was not designed to explore why nutrition practices were not meeting recommended guidelines for energy and protein intakes, which warrants further study.

Overall, the 11 participating sites had good adherence (>80%) to guidelines for prescribing energy (average 27–30 kcal/kg/day) and protein (average 1.3–1.5 g protein/kg/day). Achieving these nutritional targets was a challenge; energy intakes for patients with HN cancers were consistently between 20 and 25 kcal/day and protein intakes were ~1.1 g/kg/day, whereas patients with ESO cancers intake declined after the baseline period to an average of ~18 kcal/kg/day and ~0.8 g protein/kg/day, which are suboptimal for weight maintenance. Although the route of nutrition prescribed (EN > oral intake only > oral with ONS) helped patients get closer to their target nutritional intakes, these targets were not met and weight loss was not attenuated in either group. In studies of patients undergoing treatment for HN cancers, attenuation of weight and skeletal muscle loss was observed with energy and protein intakes of >30 kcal/kg/day and >1.0 g/kg/day, respectively [41,42]. The energy and protein intakes reported here are similar to those reported by Bargetzi et al. [43]. In that study, cancer patients at increased risk of malnutrition were randomized to receive individualized nutrition support delivered by a dietitian (e.g., oral intake + ONS; EN or PN vs. oral intake only) vs. usual care without nutrition support during their hospital stay. Compared to the usual care group, the nutrition support group had higher energy (mean 20.9 kcal/kg/day vs. 16.6 kcal/kg/day) and protein (0.8 g pro/kg/day vs. 0.6 g pro/kg/day) intakes. However, intakes for both groups were suboptimal, and below European Society for Clinical Nutrition and Metabolism (ESPEN) recommendations [22]. Despite suboptimal intakes those receiving nutrition support, compared to those who did not, experienced improved functional outcomes and quality of life, demonstrating a positive effect of using person-centered nutrition support strategies delivered by a dietitian [43]. As discussed by Bozzetti [44], perhaps there is a disassociation between improvements in body weight, muscle mass and improvements in functional status and quality of life. This may be of importance when discussing goals of care with patients.

Barriers to implementation and adherence to nutrition prescriptions and interventions were not evaluated in this study, and are likely multifactorial and related to key issues at the patient, provider, and health system levels [27,45,46,47]. This includes the need for increased adoption and adherence of EBGs into practice. Key EBGs requiring further study include benefits of (1) proactive use of EN via percutaneous gastrostomy (PEG) commencing pre-cancer treatment versus use of reactive nasogastric (NG) tubes later in treatment, (2) increased intensity of nutrition intervention/dietetic counselling, e.g., weekly dietitian visits, and (3) understanding barriers to adherence to nutrition prescriptions, inclusive of the patient experience. Other related factors may include more severe disease, increased side effects from treatment, and higher symptom burden. Differences in nutrition care protocols or individual practitioners may also affect decisions regarding the timing for initiation of nutrition support. Although we could not account for the effect of treatment in our study, we recognize it to be a major contributor to the severity and type of nutrition impact symptoms patients’ experience, as well contributing to inflammation and tissue catabolism, contributing to increased weight loss.

There are limitations to this study. Data were collected by a small number of individual clinicians through quota sampling resulting in a small sample size and lack of ethnic diversity. We acknowledge a need to include ethnically diverse study participants to create study cohorts reflective of all patients who experience HN and ESO cancers. While the inclusion of 11 international sites was a strength, it highlights the variation in nutrition practices around the world, which can differ according to center and individual clinician. We acknowledge there is variation in the methods and tools used across and within nutrition care practices. In addition, longitudinal data were collected at irregular, subject-specific intervals, as occurs in clinical practice, which resulted in patient attrition from later follow-up and a small sample size as the study progressed over time. Our study was not powered to evaluate the impact of individual level nutrition care practices at each cancer center or to evaluate impact of treatment modality on clinical outcomes. We acknowledge these factors may have contributed to a selection bias, and limit the generalizability of our findings.

In our earlier paper, Findlay et al. [37], we reported that although RDs’ nutrition care practices generally adhere to EBGs, there are significant gaps that exist when translating to the patient level. We chose to look more closely at those gaps in the current study. What is novel about this paper is that it describes what is actually being done for patients with HN and ESO cancers in clinical practice in terms of nutrition screening, route of nutrition, adequacy of energy and protein intakes, and changes in body weight that occur. For example, we found that although RDs are prescribing energy and protein intakes according to EBGs, patients are not meeting these targets despite using different routes of feeding and continue to lose weight. From the literature, it is acknowledged that weight loss (pre-treatment and/or during treatment) is associated with poor clinical and patient centered outcomes [5,48]. EBGs are created to help providers optimize nutrition care delivery, and are based on studies that demonstrate a positive impact on clinical, cost, and patient-centered outcomes [49,50]. Our study identifies a gap between prescribing and adherence to EBG as an area of focus for future work.

The descriptive analysis of nutrition care practices presented therefore identifies potential windows of opportunity highlighting time points along the treatment trajectory where nutrition support may be required for patients with HN (e.g., 2 months into treatment) and ESO (e.g., 2–4 months into treatment) cancers, and identifies areas where increased adoption and adherence to EBGs is necessary. These windows require further investigation, and may inform the design of future studies to identify additional care needs and resources unique to patients with HN and ESO cancers worldwide.

## Figures and Tables

**Table 1:** Patient characteristics at baseline.

	Head & Neck Cancers (*n* = 119) *n* (%)	Esophageal Cancers (*n* = 51) *n* (%)
**Demographics**		
Centre (Country—City)		
Australia—Brisbane	10 (8.4%)	0 (0.0%)
Australia—Sydney	20 (16.8%)	0 (0.0%)
Canada—Calgary ^1^	21 (17.6%)	10 (19.6%)
Canada—Edmonton ^1^	20 (16.8%)	18 (35.3%)
Italy—Rome	19 (16.0%)	0 (0.0%)
Netherlands—Amsterdam	20 (16.8%)	23 (45.1%)
USA—Sacramento	9 (7.6%)	0 (0.0%)
Age, years		
median (Q1, Q3)	62 (58, 69)	65 (56, 71)
Sex		
Male	93 (78.2%)	41 (80.4%)
Female	26 (21.8%)	10 (19.6%)
Ethnicity		
Caucasian	111 (93.3%)	50 (98.0%)
First Nations	1 (0.8%)	0 (0.0%)
Hispanic	2 (1.7%)	0 (0.0%)
Asian	3 (2.5%)	1 (2.0%)
East Indian	1 (0.8%)	0 (0.0%)
Other	1 (0.8%)	0 (0.0%)
Current Smoker		
Yes	44 (37.0%)	13 (25.5%)
No	75 (63.0%)	38 (74.5%)
Alcohol use		
Yes	47 (39.5%)	9 (17.6%)
No	72 (60.5%)	42 (82.4%)
ECOG Performance Status		
0	78 (65.5%)	32 (62.7%)
1	32 (26.9%)	16 (31.4%)
2	6 (5.0%)	3 (5.9%)
3	3 (2.5%)	0 (0.0%)
BMI, kg/m^2^ median (Q1, Q3)	25.8 (23.0, 30.0)	27.5 (24.5, 31.0)
BMI Class		
<18.5	6 (5.0%)	1 (2.0%)
18.5–24.9	46 (38.7%)	13 (25.5%)
25.0–29.9	37 (31.1%)	21 (41.2%)
≥30.0	30 (25.2%)	16 (31.4%)
Cancer Stage		
1	5 (4.2%)	4 (7.8%)
2	8 (6.7%)	13 (25.5%)
3	18 (15.1%)	15 (29.4%)
4 (Any)	74 (62.2%)	4 (7.8%)
Could not assess stage	7 (5.9%)	9 (17.7%)
Not staged	7 (5.9%)	4 (7.8%)
Missing	0 (0%)	2 (3.9%)
Tumor Site—Head & Neck		
Primary unknown	3 (2.5%)	
Hypopharynx	10 (8.4%)	
Larynx	22 (18.5%)	
Nasopharynx	5 (4.2%)	
Oral cavity	32 (26.9%)	
Oropharynx	39 (32.8%)	
Other	3(2.5%)	
Salivary gland	5 (4.2%)	
Treatment Modality ^2^	*n* = 100	*n* = 51
None	5 (5.0%)	4 (7.8%)
Chemotherapy—definitive	0 (0.0%)	2 (3.9%)
Chemotherapy—adjuvant	0 (0.0%)	1 (2.0%)
Radiotherapy—definitive	15 (15.0%)	0 (0.0%)
Surgery	10 (10.0%)	2 (3.9%)
Chemoradiotherapy—definitive	54 (54.0%)	7 (13.7%)
Surgery + adj/neoadj RT	7 (7.0%)	0 (0.0%)
Surgery + adj/neoadj CRT	9 (9.0%)	35 (68.6%)

Abbreviations: adj, adjuvant; ECOG, Eastern Cooperative Group performance Status; neoadj, neoadjuvant; RT, radiotherapy; CRT, chemoradiotherapy. ^1^ 3 participating sites each from Edmonton and Calgary. ^2^ Treatment modality was not captured from 19 patients from one site (Rome, Italy).

**Table 2:** Results from the PG-SGA SF over time.

	Head & Neck Cancers				Esophageal Cancers			
	Baseline	2 Months	4 Months	6 Months	Baseline	2 Months	4 Months	6 Months
	*n* = 116	*n* = 104	*n* = 105	*n* = 90	*n* = 44	*n* = 38	*n* = 38	*n* = 36
PG-SGA total score, median (Q1, Q3)	5 (1, 10)	12 (7, 18)	7 (4, 10)	6 (2, 11)	8 (4, 11)	7 (2, 15)	9 (6, 10)	5 (2, 9)
PG-SGA Triage, *n*(%)								
0–1 ^1^	33 (28)	8 (8)	13 (13)	15 (17)	4 (9)	6 (16)	1 (3)	4 (11)
2–3 ^2^	13 (11)	6 (6)	12 (12)	15 (17)	6 (14)	5 (13)	5 (13)	6 (17)
4–8 ^3^	33 (28)	21 (20)	39 (37)	27 (30)	14 (32)	11 (29)	12 (32)	17 (47)
≥9 ^4^	37 (32)	69 (66)	40 (38)	32 (36)	20 (45)	16 (42)	20 (53)	9 (25)

Abbreviations: BMI, body mass index; *n*, number; NIS, nutrition impact symptom; PG-SGA SF, patient generated-subjective global assessment short form; RD, registered dietitian; Q, quartile. ^1^ no intervention, re-assess regularly, ^2^ patient & family education; pharmacological intervention as indicted by symptoms, ^3^ intervention by RD, and nurse or physician as indicated by symptoms, ^4^ critical need of symptom management and nutrition intervention.

## Data Availability

The data presented in this study may be available from the corresponding author. The data are not publicly available due to privacy and ethical reasons.

## Supplementary Materials

The following supporting information can be downloaded at: https://www.mdpi.com/article/10.3390/nu14245272/s1, Table S1: Number of patients with complete PG-SGA SF, route of nutrition, and energy and protein intake data per audit period.
