# Supplementary material for: A Multi-Site, International Audit of Malnutrition Risk and Energy and Protein Intakes in Patients Undergoing Treatment for Head Neck and Esophageal Cancer: Results from INFORM
Source: Nutrients. 2022 Dec 10;14(24):5272. doi: 10.3390/nu14245272 (PMC9785112; doi:10.3390/nu14245272)
Supplement: Supplementary file 1 [file nutrients-14-05272-s001.zip › nutrients-2073009-supplementary.pdf]

Supplementary Table S1: Number of patients with complete PG-SGA SF, route of nutrition, and energy and protein intake data per audit period.

Type of Cancer	Audit Period	Patients with PG-SGA SF recorded	Patients with nutrition route recorded	Patients with energy and protein intakes recorded	Patients with PG-SGA SF and energy and protein intakes recorded
HN	Any*	119	103	81	78
	Baseline	116	97	70	68
	Month 2	104	84	64	57
	Month 4	105	57	41	38
	Month 6	90	27	18	16
ESO	Any*	49	50	50	49
	Baseline	44	50	48	42
	Month 2	38	37	36	28
	Month 4	38	39	40	32
	Month 6	36	21	22	15

Abbreviations: ESO, esophageal cancer; HN, head and neck cancer; PG-SGA SF, patient generated-subjective global assessment short form
